# Protocol for 3D Bioprinting a Co-culture Skin Model Using a Natural Fibrin-Based Bioink as an Infection Model

**DOI:** 10.21769/BioProtoc.5380

**Published:** 2025-07-20

**Authors:** Giselle Y. Díaz, Madeleine A. Perry, Laura S. Cárdenas, Victor A. Da Silva, Kali Scheck, Silken A. Tschofen, Stephen W. Tuffs, Stephanie M. Willerth

**Affiliations:** 1 Department of Mechanical Engineering, University of Victoria, Victoria, BC, Canada; 2 School of Engineering and Sciences, Tecnológico de Monterrey, Zapopan, Jalisco, Mexico; 3 Axolotl Biosciences, Victoria, BC, Canada; 4 Department of Biochemistry and Microbiology, Victoria, BC, Canada; 5 Division of Medical Sciences, University of Victoria, Victoria, BC, Canada; 6 Biomedical Engineering Program, University of Victoria, Victoria, BC, Canada; 7 Centre for Advanced Materials and Technology, University of Victoria, Victoria, BC, Canada; 8 School of Biomedical Engineering, University of British Columbia, Vancouver, BC, Canada

**Keywords:** 3D bioprinting, Skin model, Epidermal keratinocytes, Dermal fibroblasts, Co-culture, Microbiome, Bacteria, Fibrin-based bioink, Co-infection

## Abstract

The skin microbiome, a diverse community of microorganisms, plays a crucial role in maintaining skin health and homeostasis. Traditional studies have relied on two-dimensional (2D) models, which fail to recreate the complex three-dimensional (3D) architecture and cellular interactions of in vivo human skin, and animal models, which have species-specific physiology and accompanying ethical concerns. Consequently, both types of models fall short in accurately replicating skin physiology and understanding its complex microbial interactions. Three-dimensional bioprinting, an advanced tissue engineering technology, addresses these limitations by creating custom-designed tissue scaffolds using biomaterial-based bioinks containing living cells. This approach provides a more physiologically relevant 3D structure and microenvironment, allowing the incorporation of microbial communities to better reflect in vivo conditions. Here, we present a protocol for 3D bioprinting an in vitro skin infection model by co-culturing human keratinocytes and dermal fibroblasts in a high-viscosity, fibrin-based bioink to mimic the dermis and epidermis. The bioprinted skin tissue was co-infected with *Staphylococcus aureus* and *Staphylococcus epidermidis* to mimic bacterial skin disease. Bacterial survival was assessed through colony-forming unit enumeration. By incorporating bacteria, this protocol offers the potential to serve as a more representative in vivo 3D bioprinted skin infection model, providing a platform to study host–microbe interactions, immune responses, and the development of antimicrobial therapeutics.

Key features

• This protocol provides a detailed description of the cell culture process for both keratinocyte and fibroblast cells.

• This protocol outlines step-by-step preparation of the high-viscosity fibrin bioink and chemical crosslinker.

• The protocol uses an extrusion-based bioprinter, with an easy-to-follow methodology that clarifies the printing details, including the incorporation of skin cells into the bioink.

• This protocol details how the bacteria are inoculated into the construct to achieve the co-infection 3D skin model.

## Graphical overview


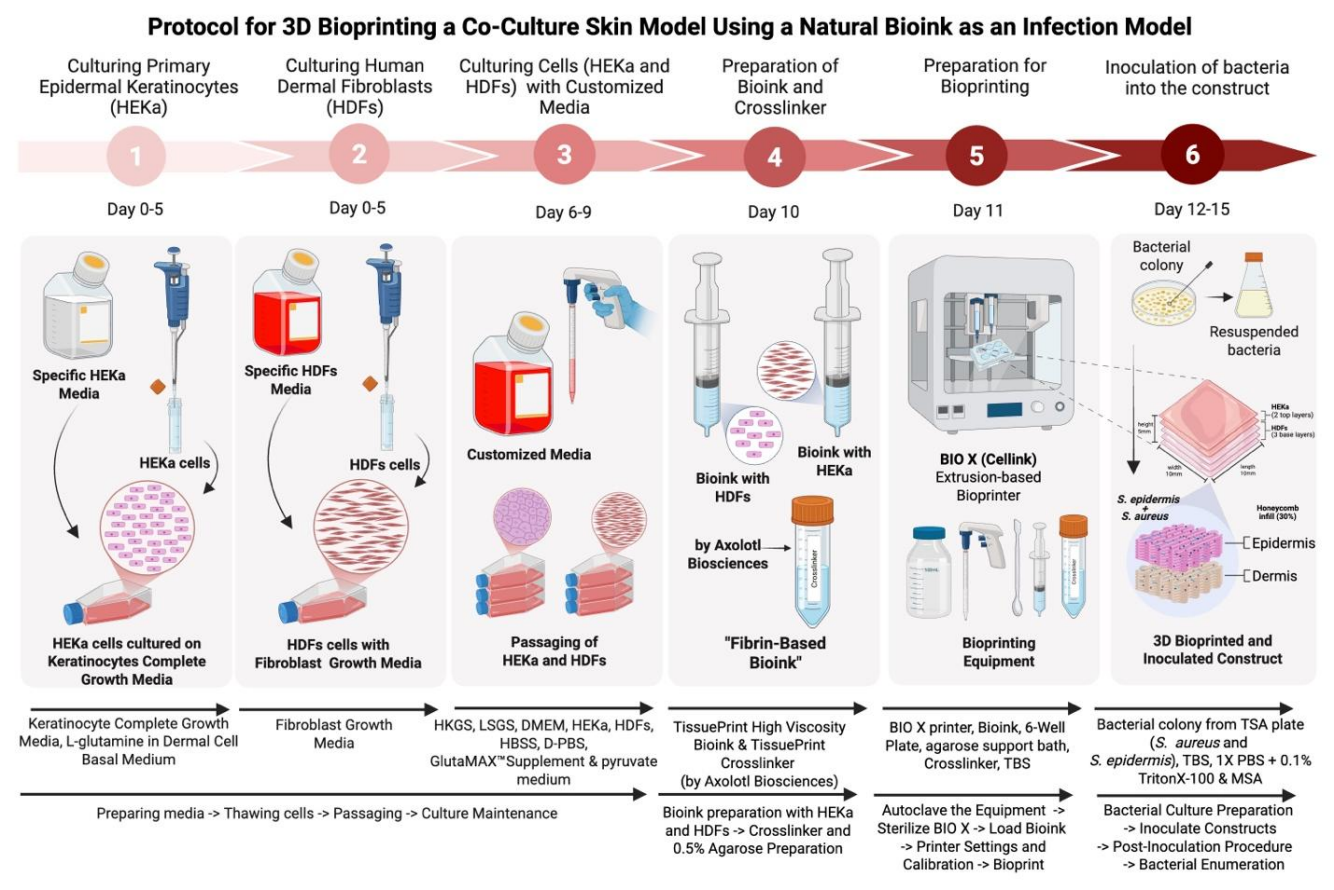


## Background

The skin of humans and other mammals hosts a highly diverse microbial community that plays a critical role in maintaining skin homeostasis and defending against pathogens [1,2]. Commensal microorganisms engage in protective innate and adaptive immune mechanisms and act as a first line of defense against external stressors such as pH fluctuations and temperature changes [3,4]. For example, *Staphylococcus epidermidis (S. epidermidis)*, a coagulase-negative staphylococci (CoNS) species, secretes extracellular serine protease (Esp), which inhibits *Staphylococcus aureus (S. aureus)* colonization by degrading proteins essential for adhesion and biofilm formation. Additionally, *S. epidermidis* stimulates keratinocytes to release antimicrobial peptides (AMPs), such as β-defensins and cathelicidin, which undermine *S. aureus* biofilms [5].

The skin maintains a delicate equilibrium between commensal microbes, skin cells, and immune cells, dynamically adapting to environmental changes to preserve barrier function [6]. Disruptions to this balance—caused by environmental stressors, infectious agents, or genetic mutations—can lead to dysregulation of the host-microbiome equilibrium, increasing susceptibility to skin infections and chronic inflammatory conditions such as atopic dermatitis (AD) [7,8]. Despite the critical role of the skin microbiome in health and disease, the precise mechanisms by which it maintains barrier homeostasis and defends against pathogens remain poorly understood [9].

Advanced in vitro models that accurately replicate the complex skin microenvironment and microbiome are needed to understand these interactions and competition between the skin microbiome, invading opportunistic pathogens, and organisms that do both (pathobionts) [10]. Traditional two-dimensional (2D) cell cultures and animal models have significant limitations. While t2D cultures lack physiological complexity, animal models are hindered by species-specific differences and ethical concerns [8]. Three-dimensional (3D) bioprinting has emerged as a powerful alternative, enabling the creation of in vitro skin models that more closely mimic human in vivo conditions. Using bioinks—biocompatible hydrogels containing living cells—3D bioprinting allows for precise spatial organization and the formation of tissue-like structures, including multi-layered constructs that replicate the dermis and epidermis [11,12]. These models support cell proliferation, differentiation, and physiologically accurate cell interactions, making them ideal for studying bacterial infections and host responses [13,14].

Recent studies have used 3D bioprinted skin models to investigate bacterial infections. For example, Villata et al. developed a 3D dermis–epidermis model using gelatin methacryloyl (GelMA) to study wound healing and bacterial infections [15]. However, a recent study by Rashad et al. highlighted that the high viscosity of GelMA in 3D bioprinting can negatively impact critical factors such as cell viability and morphology [16]. Our protocol employs a fibrin-based bioink, which offers superior biocompatibility and promotes better cell survival and structural integrity [17]. Similarly, Kohda et al. used a 3D epidermal model to study the growth dynamics of *S. epidermidis* and *S. aureus*, demonstrating that commensal bacteria can inhibit pathogenic growth and reduce epidermal cytotoxicity [18].

While these studies provide valuable insights, they are limited by their focus on single-layer models or the use of less physiologically relevant materials. Our protocol addresses these limitations by presenting a method for 3D bioprinting a multi-layered human skin model using a fibrin-based bioink. The model incorporates both keratinocytes and fibroblasts to replicate the dermal and epidermal layers of human skin. By co-infecting the model with *S. epidermidis* and *S. aureus*, we aim to study the interactions between these pathobionts that cohabitate the skin, in a physiologically relevant context. Bacterial survival is quantified through colony-forming unit (CFU) enumeration over a 72-h period. This approach provides a platform for investigating host–microbe interactions, immune responses, and the development of antimicrobial therapies.

The fibrin-based bioink used in our protocol, TissuePrint, has been successfully employed in 3D bioprinting of neural tissue models, demonstrating its versatility and biocompatibility [19–22]. Unlike previous protocols, ours focuses on co-culturing human skin cells to mimic the layered structure of human skin and introduces a detailed method for infecting 3D bioprinted constructs with skin bacteria. This makes our protocol particularly valuable for generating infection models and studying skin microbiome dynamics.

Despite its advantages, our model has some limitations. The lack of vascularization restricts its ability to fully replicate in vivo cellular responses and bacterial interactions. Additionally, while CFU enumeration provides insights into bacterial survival, it does not capture spatial distribution or the precise role of bacteria in skin barrier function. Future studies could incorporate assays such as lactate dehydrogenase (LDH) release to assess keratinocyte cytotoxicity and further elucidate the protective role of commensal bacteria [18]. Another limitation is the degradation of constructs during extended culture periods, which could be addressed by optimizing culture conditions to allow for epidermal maturation, as demonstrated by Liu et al. [23]. In conclusion, our protocol offers a physiologically accurate 3D bioprinted skin model that incorporates the skin microbiome, providing a valuable tool for studying host-microbe interactions, skin infections, and the development of novel therapies. While challenges remain, this model represents a significant advancement over traditional 2D cultures and animal models, with broad applications in dermatological research.

## Materials and reagents

**Biological materials**

1. Primary epidermal keratinocytes (HEKa) (ATCC, catalog number: PCS-200-011)

2. Human dermal fibroblasts (HDFs) (Cell Applications Inc., catalog number: 106-05a)

3. *Staphylococcus epidermidis* strain RP62a (BEI Resources, catalog number: NR-45879) [24]

4. *Staphylococcus aureus* strain MW2 (BEI Resources, catalog number: NR-46054) [25]

**Reagents**

1. Dermal cell basal media (ATCC, catalog number: PCS 200-030)

2. Keratinocyte Growth kit (ATCC, catalog number: PCS 200-040), containing: bovine pituitary extract (BPE), 2.0 mL, 0.4%; rh TGF-a, 0.5 mL, 0.5 ng/mL; L-Glutamine, 15.0 mL, 6 mM; hydrocortisone hemisuccinate, 0.5 mL, 100 ng/mL; rh insulin, 0.5 mL, 5 μg/mL; epinephrine, 0.5 mL, 1.0 μM; Apo-Transferrin, 0.5 mL, 5 μg/mL

3. Fibroblast growth media (Cell Applications Inc., catalog number: 116-500)

4. Dulbecco’s modified Eagle medium (DMEM) high glucose, GlutaMAX^TM^ supplement, pyruvate (Thermo Fisher Scientific, catalog number: 10569-010)

5. Human keratinocyte growth supplement (HKGS) (Thermo Fisher Scientific, catalog number: S001)

6. Low serum growth supplement (LSGS) (Thermo Fisher Scientific, catalog number: S00310)

7. Trypan blue (Thermo Fisher Scientific, catalog number: 15250-061)

8. Trypsin-ethylenediaminetetraacetic acid (EDTA) (for fibroblasts) (Cell Applications Inc., catalog number: 070-100)

9. Trypsin-ethylenediaminetetraacetic acid (EDTA) (for keratinocytes) (ATCC, catalog number: PCS 999 003)

10. Trypsin neutralizing solution (for fibroblasts) (Cell Applications Inc., catalog number: 070-100)

11. Fetal bovine serum (FBS) (R&D Systems, catalog number: S12450)

12. Dulbecco’s phosphate-buffered saline (DPBS) (R&D Systems, catalog number: B30250)

13. Hank’s balanced salt solution (HBSS) (Thermo Fisher Scientific, catalog number: 14-175-095)

14. Phosphate buffer solution (PBS) (Thermo Fisher Scientific, catalog number: 10010-023)

15. Tris hydrochloric acid (Tris HCl) (VWR Chemicals, catalog number: 22J175305)

16. Tris(hydroxymethyl)aminomethane (Tris base) (Thermo Fisher Scientific, catalog number: 198252)

17. Sodium chloride (NaCl) (Acros Organic, catalog number: A0425202)

18. Potassium chloride (KCl) (Caledon, catalog number: 73495)

19. Distilled water

20. Froggarose LE (FroggaBio, catalog number: A87-500G)

21. Triton X-100 (Sigma-Aldrich, catalog number: 9036-19-5)

22. Ethanol, 95% (Greenfield Global, catalog number: 9036-19-5)

23. TissuePrint (Axolotl Biosciences, catalog number: AX001)

24. TissuePrint Crosslinker (Axolotl Biosciences, catalog number: AX002)

25. Mannitol salt agar (BD, catalog number: BD 211407)

26. Tryptic soy agar (Millipore Sigma, catalog number: 22091)

27. Tryptic soy broth (Millipore Sigma, catalog number: 22092)

**Solutions**

1. Tris-buffered saline (TBS) solution (see Recipes)

2. 0.1% Triton-X (see Recipes)

3. 70% ethanol solution (see Recipes)

4. 5% trypsin neutralizing solution (for keratinocytes) (see Recipes)

**Recipes**

**1. Tris-buffered saline (TBS) solution**

To make 4 L of TBS solution, dissolve the following reagents in distilled water while stirring on a magnetic stir plate. Adjust the pH to 7.7. The prepared TBS is kept at room temperature and is disposed of after use in this protocol.

**Table:** 

Reagent	Final concentration	Quantity or volume
Tris HCl	27.7 mM	17.44 g
Tris base	5.3 mM	2.56 g
NaCl	136.9 mM	32 g
KCl	2.7 mM	0.8 g
Distilled water	n/a	4 L

**2. 0.1% Triton-X solution**

To prepare 25 mL of 0.1% Triton-X, mix the following reagents in a 50 mL conical tube and vortex until fully dissolved. Triton X-100 (100%) can be stored at room temperature for 24 months.

**Table:** 

Reagent	Final concentration	Quantity or volume
PBS	n/a	24.975 mL
Triton X-100 (100%)	0.1%	25 μL

**3. 70% ethanol solution**

To prepare 5.43 L of 70% ethanol solution, mix the following reagents in a large plastic container. The prepared ethanol is kept at room temperature until it is fully consumed.

**Table:** 

Reagent	Final concentration	Quantity or volume
Ethanol (95%)	70%	4 L
Distilled water	n/a	1.43 L

**4. 5% trypsin neutralizing solution (for keratinocytes)**

To prepare 10 mL of a 5% trypsin neutralizing solution, mix the following reagents in a 50 mL conical tube inside a biological safety cabinet (BSC). The prepared solution is stored at 4 °C for up to 2 weeks.

**Table:** 

Reagent	Final concentration	Quantity or volume
FBS	5%	500 μL
PBS	n/a	9.5 mL

**Laboratory supplies**

1. T-75 Corning flasks (Corning, catalog number: CLS430641U-100EA)

2. Serological pipettes 5 mL (Falcon, catalog number: 357543)

3. Serological pipettes 10 mL (Fisherbrand, catalog number: 13-678-11)

4. Serological pipettes 25 mL (Fisherbrand, catalog number: 13-678-11E)

5. 5 mL syringe (Terumo, catalog number: SS-05L)

6. 10 mL syringe (BD, catalog number: 302995)

7. 100 mL graduated cylinder

8. 250 mL glass beaker

9. Glass pipette

10. Magnetic stir plate and stir bar

11. 50 mL conicals (Fisherbrand, catalog number: 05-539-13)

12. 15 mL conicals (Falcon, catalog number: 352196)

13. Autoclavable containers

14. Reusable glass media bottles with cap (100 mL, 250 mL, 1000 mL) (Fisherbrand, catalog number: FB-800-100)

15. 0.2 μm cellulose acetate sterile syringe filter (Sartorius, catalog number: 17823----------K)

16. 0.2 μm PTFE syringe filter (VWR, catalog number: 76479-044)

17. Parafilm (Amcor, catalog number: PM-996)

18. 4 L container (VWR, catalog number: ca7350-022)

19. P1000, P200, and P20 pipettes (Axygen, catalog numbers: BT-1000-R-S, BT-250-R-S, BT-50-R-S)

20. Pipette tips (20 μL, 200 μL, and 1000 μL)

21. pH meter

22. Conical rack

23. Metal spatula

24. 500 mL beaker

25. 200 mL beaker

26. 250 mL beaker

27. 6-well cell culture plate (Cellstar, catalog number: 657160)

28. 12-well cell culture plate (Cellstar, catalog number: 662160)

29. Metal Luer locks

30. Sterile empty cartridges with end and tip caps, 3 mL (CELLINK, catalog number: CSC010311101)

31. Sterile standard blunt needles 22G (CELLINK, catalog number: NZ5220255001)

32. Kimwipes

33. Waste container

## Equipment

1. BIO X printer (CELLINK, catalog number: D16110020717, fitted with a 3 mL pneumatic printhead bioprinting nozzle

2. Microscope (Leica, model: DMI3000B)

3. FormaTM Steri-CycleTM CO_2_ incubator (Thermo Fisher, model: 370)

4. Biosafety Cabinet (Microzone, model: BK-2-4)

5. Water bath (VWR, catalog number: 89032-299)

6. Centrifuge 5810 R (Eppendorf, catalog number: 5811000015)

7. DeNovix CellDrop FL (FroggaBio, model: DeNovix CellDrop FL)

8. Autoclave (Market Forge, model: STM-EL)

9. Cytation 5 Cell Imaging Multimode Reader (BioTek/Agilent, model: Cytation 5)

## Software and datasets

1. QCapture Software (QImaging, version 2.9.12), QImaging (https://www.qimaging.com)

2. Excel (Microsoft, latest version), Microsoft (https://www.microsoft.com/en-us/microsoft-365/excel)

3. ImageJ (National Institutes of Health, version 1.52a), NIH (https://imagej.nih.gov/ij/); open source

4. Clipchamp Software (Microsoft, latest version), Microsoft (https://www.clipchamp.com)

5. Fliki AI (Fliki, latest version), Fliki (https://www.fliki.ai)

6. GraphPad Prism 10 (GraphPad Software, version 10), GraphPad Software (https://www.graphpad.com)

## Procedure

The procedures for handling primary epidermal keratinocytes are based on the guidelines provided by the American Type Culture Collection (ATCC), as outlined in their Primary Epidermal Keratinocyte Product Sheet, last updated in 2024 [26]. Additionally, the procedures for handling human dermal fibroblasts are based on the Cell Applications Inc. guidelines for Culturing Human Dermal Fibroblasts [27].

**A. Culturing primary epidermal keratinocytes**

**A1. Preparing the keratinocyte complete growth media**

Timing: 2 h

Conditions: Room temperature (RT), light sensitive, sterile

1. Allow the Keratinocyte Growth kit to thaw at 4 °C for 1 h.

2. Warm the L-glutamine in a bead bath at 35 °C and gently swirl the bottle to ensure complete dissolution.

*Notes:*

*1. The L-glutamine should dissolve completely and turn transparent.*

*2. For complete dissolution of L-glutamine, the solution must turn transparent. If it does not, allow more time for it to dissolve, or use a fresh stock of L-glutamine.*

3. Inside the Biological Safety Cabinet (BSC), add the indicated volumes of each component from the Growth kit to 485 mL of dermal cell basal medium using a serological pipette.

4. Mix the medium by carefully resuspending it 15–20 times with a 10 mL pipette.

5. Store the prepared media at 4 °C for a maximum of 30 days.

**A2. Procedure for thawing cells**

Timing: 45 min + 30 min incubation

Conditions: RT, light sensitive, sterile

1. Let the keratinocyte complete growth media reach RT by placing the bottle in a dark location for 30 min to 1 h prior to use.

2. Calculate the required volume of cells from the cryovial to achieve the desired seeding density (5,000–8,000 cells/cm^2^ for HEKa cells).

Example calculation for a T-75 flask with 500,000 cells:

If the cryovial contains 2 million cells in 1 mL of freezing solution, calculate the volume needed using Equation 1.

$$\frac{Number\;of\;cells}{Concentration\;of\;cells\;(cells/mL)}=Volume\;of\;cell\;solution\;required.$$

$\frac{500,000cells}{2,000,000cells/mL}=0.25\;mL\;of\;cell\;solution$ **Equation 1**

3. Disinfect the flasks and media with 70% ethanol and place them in the BSC.

*Note: All subsequent handling of media and cells should be performed inside the BSC to maintain aseptic conditions.*

4. Add 15 mL of keratinocyte complete growth media at RT to the T-75 flask.

5. Place the flasks in a 37 °C, 5% CO_2_ incubator for 30 min.

6. Thaw the cryovial by gently warming it in your hands until no ice remains.

7. Slowly add 1 mL of prewarmed media to the cryovial.

8. Gently pipette the mixture up and down 2–3 times to resuspend the cells.

**Critical:** Do not pipette up and down excessively, as this may cause cell loss or damage. Avoid introducing air bubbles into the solution.

9. Transfer the solution into a 15 mL conical tube.

10. Centrifuge the conical tube at 150× *g* for 3 min. Carefully aspirate and discard the supernatant without disturbing the cell pellet.

*Note: This step is to remove the dimethyl sulfoxide (DMSO) where the cells were cryopreserved.*

11. Add 1 mL of fresh media to the cell pellet.

12. Gently pipette up and down until the pellet is fully dispersed and the cell suspension is homogeneous.

13. In the BSC, transfer the appropriate volume of cell solution from the cryovial into the flask using a sterile pipette.

*Note: Pipette up and down in the cryovial to ensure homogeneity before transferring the cell solution.*

14. Add the appropriate volume of cell solution into the flask through the opening while holding the flask horizontally.

15. Gently rock the flask to evenly distribute the cells.

*Note: Perform 10 gentle movements in both the horizontal (T) and vertical directions (10 times each).*

16. Incubate the seeded culture flasks at 37 °C, 5% CO_2_ for 48 h.

See Troubleshooting 1.

**A3. Passaging procedure**

Timing: 1–2 h

Conditions: RT, light sensitive, sterile

1. Assess the cell confluence of the flasks using a microscope at 10× magnification.

*Notes:*

**Figure 1.**
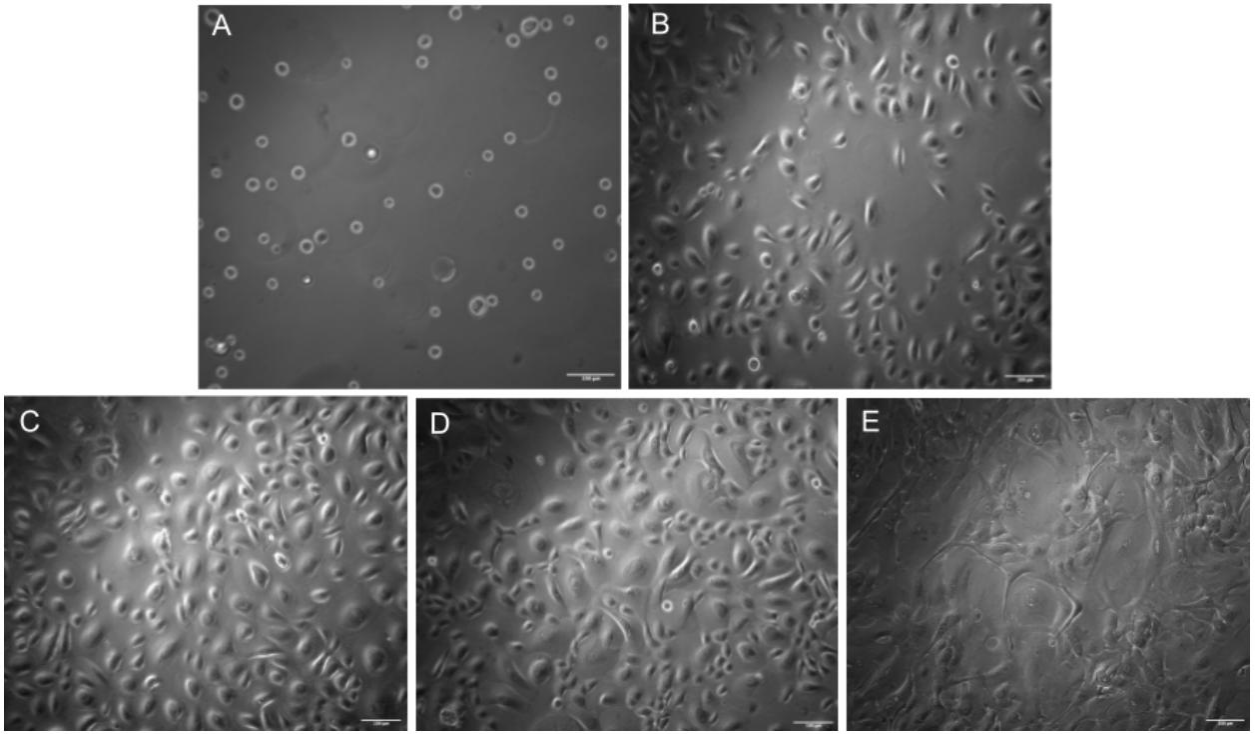
Brightfield microscopy images of keratinocytes cultured for 8 days with the addition of customized media on Day 6. Images were taken at 10× resolution. (A) Day 0: Cells immediately after seeding. (B) Day 2. (C) Day 4. (D) Day 6: Customized media added. (E) Day 8. Scale bars: 100 μm.

*1. Ensure the cells are 70%–75% confluent before passaging. Refer to Figure 1 to visualize the cellular morphology of HEKa throughout the culture period, leading up to bioprinting.*

*2. Visual assessment is sufficient.*

2. Allow keratinocyte complete growth media, DPBS, trypsin-EDTA, and trypsin neutralizing solution to reach RT.

*Note: Store the media in a dark place.*

3. Sterilize the work area with 70% ethanol and place materials in the BSC.

4. Remove the used media from each flask using a serological pipette and dispose of it into a liquid waste beaker.

*Note: After working in the BSC, dispose of liquid waste by adding 3%–6% sodium hypochlorite solution (household bleach) to neutralize it, then pour down the sink within 30 s.*

5. Rinse the cell layer with 5 mL of DPBS.

*Note: Rinse twice if necessary to remove any leftover media and cell debris.*

6. Remove the DPBS from the flask using a serological pipette.

7. Prewarm the trypsin-EDTA solution at RT.

8. Add 5 mL of prewarmed trypsin-EDTA solution to each flask.

9. Gently rock the flask for up to 2 min to ensure the trypsin-EDTA solution evenly covers the entire cell culture surface.

10. Remove 4 mL of the solution from the flask with a serological pipette and dispose of it into a liquid waste beaker.

*Note: It is essential to prevent overexposure of cells to trypsin to preserve cell viability. A short contact/exposure time (e.g., less than 2 min) is highly recommended.*

11. Observe the cells under a microscope at 10× magnification.

*Note: Cells should begin to round and detach within 10 min.*

See Troubleshooting 2.

12. Gently tap the flask's base to help detach the cells.

13. Once cells are detached, add an equal volume of trypsin neutralizing solution to neutralize the trypsin.

14. Transfer the cell solution into a sterile, labeled 50 mL conical tube.

15. Confirm under the microscope that all cells have detached.

*Note: If over 20% of cells remain, repeat the procedure until proper detachment is achieved.*

16. Centrifuge the cells at 150× *g* for 5 min, disinfect the conical tube with 70% ethanol, and place it in a conical tube rack inside the BSC.

17. Remove the supernatant with a serological pipette, leaving the cell pellet.

18. Resuspend the cell pellet in 1 mL of keratinocyte complete growth media at RT.

19. Using a sterile pipette, remove 20 μL of the cell suspension and transfer it to an Eppendorf tube.

20. Add 20 μL of Trypan blue to the Eppendorf tube and mix gently with a pipette.

21. Use a cell counter (DeNovix CellDrop FL) to count the cells.

*Note: Calculate the volume required to seed new T-75 culture flasks at 2,500–5,000 cells/cm^2^ using Equation 1.*

22. Add 15 mL of fresh keratinocyte complete growth media to each new flask and incubate for 30 min.

23. Seed the flasks with the calculated volume of cell suspension, as calculated earlier (refer to Equation 1).

*Note: Add the suspension while holding the flask horizontally.*

24. Incubate the newly seeded flasks at 37 °C, 5% CO_2_ for 24–48 h.

*Note: Decontaminated solutions and biological waste must be disposed of according to the specific regulations and guidelines of your institution.*

**A4. Culture maintenance**

Timing: 30 min

Conditions: RT, light sensitive, sterile

1. Warm the keratinocyte complete growth media to RT by placing the bottle on the benchtop in a dark location for 30 min to 1 h.

2. Remove the culture flasks from the incubator.

3. Disinfect the flasks with 70% ethanol and place them inside the BSC.

4. Remove the used media using a serological pipette.

5. Add 15 mL of fresh prewarmed keratinocyte complete growth media to each T-75 flask.

6. Incubate the flasks and assess confluence after 48 h.

7. Repeat the media change every 48 h if the cells are not ready to passage.

8. Passage the cells once they reach 70%–75% confluence.

**B. Culturing human dermal fibroblasts**

**B1. Procedure for thawing cells**

Timing: 45 min + 30 min incubation

Conditions: RT, light sensitive, sterile

1. Allow the cryovial containing fibroblast cells to thaw by gently warming it in your hands until no ice remains.

2. Calculate the volume required from the selected cryovial to be transferred into the flasks to achieve the desired seeding density. For HDFs, the initial seeding density can be between 2,500 and 5,000 cells per cm^2^. **For example**, to seed a T-75 flask with 500,000 cells from a cryovial containing 2 million cells in 1 mL of freezing solution, use Equation 1 to calculate the required volume.

**Critical:** Ensure accurate calculations to achieve the desired seeding density.

3. Slowly add 1 mL of prewarmed media to the cryovial.

4. Gently pipette the mixture up and down 2–3 times to resuspend the cells.

**Critical:** Do not pipette up and down excessively, as this may cause cell loss or damage. Avoid introducing air bubbles into the solution.

5. Transfer the solution into a 15 mL conical tube.

6. Centrifuge the conical tube at 220× *g* for 3 min. Carefully aspirate and discard the supernatant without disturbing the cell pellet.

*Note: This step is to remove the DMSO, which is added when the cells are cryopreserved.*

7. Add 1 mL of fresh media to the cell pellet.

8. Gently pipette up and down until the pellet is fully dispersed and the cell suspension is homogeneous.

9. Disinfect the flasks and the media with 70% ethanol and place them inside the BSC.

10. Add 15 mL of fresh fibroblast growth media to each T-75 flask and incubate them for 30 min at 37 °C, 5% CO_2_.

**Pause point:** The flasks can remain in the incubator here while you complete the thawing process.

11. Transfer the appropriate volume of thawed cell solution from the 15 mL conical tube into the flask using a sterile pipette.

*Note: Pipette up and down in the growth media several times to ensure all cells are transferred.*

12. Incubate the seeded culture flasks at 37 °C, 5% CO_2_ for 48 h.

See Troubleshooting 1.

**B2. Passaging procedure**

Timing: 1–2 h

Conditions: RT, light sensitive, sterile

1. Assess the cell confluence of the flasks under a microscope. Cells should be 80% confluent for passaging. This can be estimated visually.

2. Allow the fibroblast growth medium, HBSS, trypsin-EDTA, and trypsin neutralizing solution to reach RT.

*Note: During this step, ensure the media is stored in a dark place.*

3. Place the cell culture flasks into the BSC and remove the used media from each flask using a serological pipette.

4. Rinse the flasks with 5 mL of HBSS and remove it with a serological pipette.

*Note: This step may be repeated twice to ensure complete removal of any leftover media.*

5. Prewarm the trypsin-EDTA solution at RT.

6. Add 5 mL of prewarmed trypsin-EDTA solution to each flask.

7. Gently rock the flask for up to 2 min to ensure the trypsin-EDTA solution evenly covers the entire cell culture surface.

8. Remove 4 mL of the solution from the flask with a serological pipette and dispose of it into a liquid waste beaker.

**Critical:** It is essential to prevent overexposure of cells to trypsin to preserve cell viability. A short contact/exposure time (e.g., less than 2 min) is highly recommended. Observe the cells under a microscope at 10× magnification.

*Note: The cells should begin to round and detach within 10 min.*

See Troubleshooting 2.

9. Once the cells appear rounded, gently tap the base of the flask to ensure the cells have been released from the surface of the flask.

10. Add an equal volume of trypsin neutralizing solution to neutralize the trypsin once the cells are detached.

11. Transfer the cell solution into a sterile and labeled 50 mL conical tube.

12. Confirm under the microscope that all cells have detached from the flask.

*Note: If over 20% of the cells remain attached, repeat the trypsinization procedure.*

13. Centrifuge the cells in the 50 mL conical tube at 220× *g* for 5 min. Disinfect the conical tube with 70% ethanol and place it in the conical tube rack inside the BSC.

14. Remove the supernatant with a serological pipette, leaving the cell pellet at the bottom of the tube.

15. Resuspend the cell pellet in 1 mL of fibroblast growth media in the 50 mL conical tube.

16. Using a sterile pipette, remove 20 μL of the cell suspension and place it into an Eppendorf tube.

17. Add 20 μL of Trypan blue to the Eppendorf tube and gently mix with a pipette.

18. Count the cells using a cell counter (e.g., DeNovix CellDrop FL) and calculate the volume required to seed new T-75 culture flasks at 2,500–5,000 cells/cm^2^.

19. Add 15 mL of fresh, prewarmed fibroblast growth medium to each new flask and incubate the flasks for 30 min at 37 °C, 5% CO_2_.

20. Seed the flasks with the calculated volume of cell suspension.

*Note: Pipette up and down in the growth media several times to ensure all cells are transferred.*

21. Incubate the newly seeded flasks at 37 °C, 5% CO_2_ for 48 h.

**B3. Culture maintenance**

Timing: 30 min

Conditions: RT, light sensitive, sterile

1. Allow the fibroblast growth medium to reach RT.

*Note: During this step, ensure the media is stored in a dark place. Allow the media to acclimatize for 30 min to 1 h.*

2. Remove the flasks from the incubator. Disinfect the flasks with 70% ethanol and place them inside the BSC.

3. Remove the used media from the flasks using a serological pipette.

4. Add 15 mL of fresh, prewarmed fibroblast growth medium to each T-75 flask.

5. Incubate the flasks at 37 °C, 5% CO_2_ for 48 h.

6. Repeat the media change every 48 h if the cells are not ready to passage.

7. Passage the cells once they reach 80% confluence.

**C. Culturing cells with customized media**

**C1. Preparing the customized media**

Timing: 1 h + 24 h prior

Conditions: RT, light sensitive, sterile

1. Thaw the HKGS and LSGS reagents at 4 °C.

*Note: Allow the reagents to thaw overnight before using them.*

2. Allow DMEM to reach RT.

3. In the BSC, add 5 mL of HKGS and 10 mL of LSGS to the DMEM bottle.

4. Mix the medium by gently resuspending it with a 10 mL pipette.

5. Store the prepared media at 4 °C for a maximum of 30 days.

**C2. Culturing the cells with customized media**

Timing: 2 h + 24 h prior

Conditions: RT, light sensitive, sterile

1. Remove the flasks containing cultured cells (HEKa and HDFs) from the incubator.

2. Disinfect the plate with 70% ethanol and place it in the BSC.

3. Use a serological pipette to remove the used media from each flask and dispose of it into a liquid waste beaker.

4. Prewarm the customized media to RT.

5. Add 15 mL of prewarmed customized media to each T-75 flask.

6. Return the flasks to the incubator and incubate at 37 °C and 5% CO_2_ for 48 h.

7. Proceed with the bioprinting process after the cells have adapted to the custom media for at least 48 h and have reached the desired confluence.

*Notes:*

*1. If the cells have not reached the appropriate confluence, repeat the media change every 48 h until ready for passaging and printing.*

**Figure 2.**
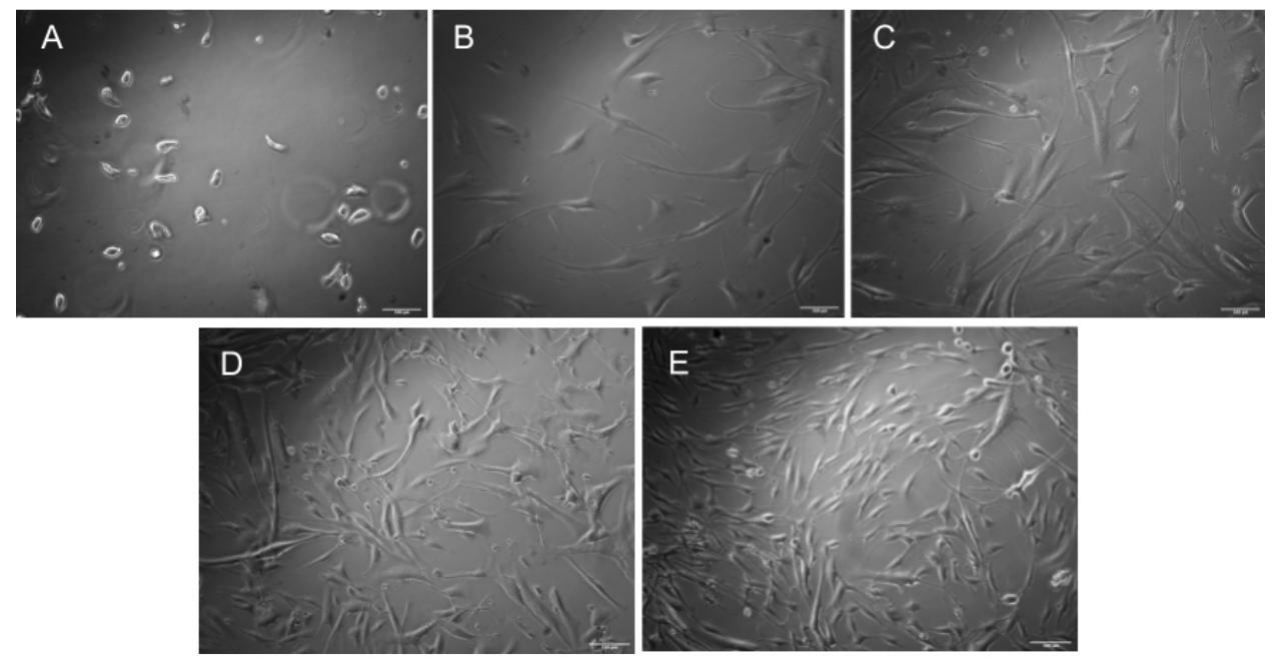
Brightfield microscopy images of human dermal fibroblasts cultured for 8 days, captured at 10× resolution. Customized media was added on Day 6. (A) Day 0: Cells immediately after seeding. (B) Day 2. (C) Day 4. (D) Day 6: Customized media added. (E) Day 8. Scale bars: 100 μm.

*2. We observe cell morphology under the microscope to determine if they are adapting to the custom media. Refer to Figure 2 to visualize the cellular morphology of HDFs throughout the culture period, leading up to bioprinting.*

**C3. Passaging procedure**

Timing: 1–2 h

Conditions: RT, light sensitive, sterile

*Note: Follow the agreed-upon steps for the appropriate passaging of HEKa and HDFs. Refer to the passaging procedure for each specific cell type.*

1. Assess the cell confluence of the flasks under a microscope.

*Note: Ensure that HEKa are 70%–75% confluent and HDFs are 80% confluent.*

2. Allow the customized media, HBSS, DPBS, trypsin-EDTA, and trypsin neutralizing solution to reach RT.

*Note: During this step, ensure the media is stored in a dark place.*

3. Place the cell culture flasks into the BSC and remove the used media from each flask using a serological pipette.

4. Rinse the flasks with 5 mL of HBSS or DPBS, depending on the cell type, and then remove the solution from the flasks using a serological pipette.

*Note: This step may be repeated twice to ensure any leftover media is removed.*

5. Prewarm the trypsin-EDTA solution at RT.

6. Add 5 mL of prewarmed trypsin-EDTA solution to each flask.

7. Gently rock the flask for up to 2 min to ensure the trypsin-EDTA solution evenly covers the entire cell culture surface.

8. Remove 4 mL of the solution from the flask with a serological pipette and dispose of it into a liquid waste beaker.

*Note: It is essential to prevent overexposure of cells to trypsin to preserve cell viability. A short contact/exposure time (e.g., less than 2 min) is highly recommended.*

9. Observe the cells under the microscope. The cells should round up and become separate from each other.

*Note: This process should take under 10 min.*

See Troubleshooting 2.

10. After the cells appear rounded, gently tap the base of the flask to ensure all cells have been released from the surface of the flask.

11. Add an equal volume of trypsin neutralizing solution, appropriate for the cell type, once the cells have detached, to neutralize the trypsin solution.

12. Transfer the cell solution into a sterile and labeled 50 mL conical tube.

13. Under the microscope, confirm that all cells have detached from the flasks.

*Note: If over 20% of the cells remain in the flask, repeat the trypsinization procedure and add these cells to the 50 mL conical tube once adequately detached.*

14. Centrifuge the cells in the 50 mL conical tube at 150× *g* for 5 min for HEKa or at 220 *g* for 5 min for HDFs.

15. Once centrifuged, disinfect the conical tube with 70% ethanol and place it in a conical rack inside the BSC.

16. Carefully remove the supernatant from the centrifuged conical tube using a serological pipette, being cautious not to disturb the cell pellet.

17. In the 50 mL conical tube, resuspend the cell pellet in 1 mL of customized media, using a 1,000 μL pipette to mix.

18. Using a sterile pipette, remove 20 μL of the cell suspension and place it into an Eppendorf tube.

19. Add 20 μL of Trypan blue to the Eppendorf tube and gently mix with a pipette.

20. Using a cell counter (e.g., DeNovix CellDrop FL), count the cells and calculate the volume required to seed new T-75 culture flasks at a density of 2,500–5,000 cells per cm^2^.

21. Add 15 mL of fresh prewarmed customized media to each new flask and incubate the flasks for 30 min at 37 °C, 5% CO_2_.

22. Seed the flasks with the calculated volume of cell suspension.

*Note: Pipette up and down in the customized media several times to ensure all cells are transferred.*

23. Incubate the newly seeded flasks at 37 °C and 5% CO_2_ for 48 h.

**D. Preparation of bioink and crosslinker**

**D1. Bioink preparation with HDFs**

Timing: 2 h (minimum) + 24 h prior

Conditions: RT, sterile

1. Prepare the TissuePrint high-viscosity bioink according to the formulation by Axolotl Biosciences.

*Note: The TissuePrint high-viscosity bioink has been used in previous bioprinting research, as shown in Chrenek et al. [19] and Perez et al. [28]. In addition, the Axolotl Biosciences website can be reviewed for more details [29].*

2. Thaw Component 1 and Component 3 of the TissuePrint kit at 4 °C.

*Note: Allow the reagents to thaw overnight before using them.*

3. Prior to mixing the bioink, passage the HDFs in accordance with the passaging procedure in culturing the cells with the customized media section.

4. Once the HDFs have been counted, proceed to calculate how much bioink needs to be prepared.

Calculation: 2 million cells per 1 mL of bioink.

**Example:** If you have 4 million HDFs and need to prepare bioink at a density of 2 million cells per 1 mL of bioink, use Equation 2.

*Notes:*

*1. Ensure that you have enough HDFs for your printing purposes. A range of 2–10 million cells per mL of bioink can be used, depending on the cell line. Accumulating enough cells may require multiple weeks of passaging.*

*2. When using the TissuePrint kit, ensure it comprises Components 1, 2, and 3 and excludes the Crosslinker component.*

$$\frac{Number\;of\;cells}{Concentration\;of\;cells\;(cells/mL)}=Volume\;of\;cell\;solution\;required$$

$\frac{2\;million\;cells}{4\;million\;cells}=\frac{1\;mL\;of\;ink}{x}\to \frac{\left(1\;mL\;of\;ink)(4\;million\;cells\right)}{2\;million\;cells}=2\;mL\;of\;Bioink$ **Equation 2**

5. Once the HDFs have been counted, centrifuge the cells at 220× *g* for 5 min.

6. After centrifugation, without disturbing the cell pellet, disinfect the conical tube with 70% ethanol and place it in a conical rack inside the BSC while other bioink components are mixed.

7. Working in the BSC, mix Component 2 with Component 1 in a 50 mL conical tube.

*Note: If Component 2 starts to clump, slowly pipette up and down using a wide-bore pipette to disperse the clump.*

8. Carefully remove the supernatant from the cell suspension without disturbing the cell pellet.

9. Add 1 mL of Component 3 at RT to the cells and resuspend by gently pipetting up and down.

*Note: Avoid harsh pipetting to prevent cell death.*

10. Add the rest of Component 3 to the cells and continue pipetting gently.

11. Slowly add the cell–Component 3 mixture into the mixture of Component 1 and Component 2 using a 1 mL pipette tip.

12. Gently mix the complete bioink solution by pipetting up and down.

*Notes:*

*1. For optimal results, use the bioink within 1 h of preparation. It is recommended to prepare small quantities of bioink (around 4 mL per batch) to minimize waste in case of issues during the bioprinting process.*

*2. While preparing additional bioink-cell bioinks, keep any previously prepared bioink on ice to minimize metabolic activity and maintain cell viability.*

**D2. Bioink preparation with HEKa**

Timing: 2 h (minimum) + 24 h prior

Conditions: RT, sterile

1. Prepare TissuePrint high-viscosity bioink according to the formulation by Axolotl Biosciences.

*Note: The TissuePrint high-viscosity bioink has been used in previous bioprinting research, as shown in Chrenek et al. [19] and Perez et al. [28]. In addition, the Axolotl Biosciences website can be reviewed for more details [29].*

2. Thaw Component 1 and Component 3 of the TissuePrint kit at 4 °C.

*Note: Allow the reagents to thaw overnight before using them.*

3. Prior to mixing the bioink, passage the HEKa cells in accordance with the passaging procedure in culturing the cells with the customized media section.

4. Once HEKa have been counted, proceed to calculate how much bioink to prepare.

Calculation: Refer to Equation 2.

*Notes:*

*1. Ensure that you have enough HEKa for your printing purposes. A range of 2–10 million cells per mL of bioink can be used, depending on the cell line. Accumulating enough cells may require multiple weeks of passaging.*

*2. When using the TissuePrint kit, ensure it comprises Components 1, 2, and 3 and excludes the Crosslinker component.*

5. Once HEKa cells have been counted, centrifuge the cells at 150× *g* for 5 min.

6. After centrifugation, without disturbing the cell pellet, disinfect the conical tube with 70% ethanol and place it in a conical rack inside the BSC while other bioink components are mixed.

7. Working in the BSC, mix Component 2 with Component 1 in a 50 mL conical tube.

*Note: If Component 2 starts to clump, slowly pipette up and down using a wide-bore pipette to disperse the clump.*

8. Carefully remove the supernatant from the cell suspension without disturbing the cell pellet.

9. Add 1 mL of Component 3 at RT to the cells and resuspend by gently pipetting up and down.

*Note: Avoid harsh pipetting to prevent cell death.*

10. Add the rest of Component 3 to the cells and continue pipetting gently.

11. Slowly add the cell–Component 3 mixture into the mixture of Component 1 and Component 2 using a 1 mL pipette tip.

12. Gently mix the complete bioink solution by pipetting up and down.

*Notes:*

*1. For optimal results, use the bioink within 1 h of preparation. It is recommended to prepare small quantities of bioink (around 4 mL per batch) to minimize waste in case of issues during the bioprinting process.*

*2. While preparing additional bioink-cell bioinks, keep any previously prepared bioink on ice to minimize metabolic activity and maintain cell viability.*

**D3. Crosslinker preparation**

Timing: 30 min

Conditions: RT, sterile

1. Prepare TissuePrint Crosslinker according to the formulation created by Axolotl Biosciences.

*Notes:*

*1. For optimal results, use 20 mL of crosslinker for each 5 mL of bioink.*

*2. Use of the TissuePrint Crosslinker has been described in the studies by Chrenek et al. [19]. and Perez et al. [28]. Additional information can be found on the Axolotl Biosciences website [29].*

2. Thaw Component A and Component B of the crosslinker at 4 °C.

*Notes:*

*1. All Crosslinker components arrive sterile.*

*2. Allow the reagents to thaw overnight before using them.*

3. Using sterile technique in a biosafety cabinet, add Component B to Component A.

4. Mix with a pipette to incorporate the two components.

*Note: The crosslinker solution has a shelf-life of one week when stored at 4 °C after preparation.*

**D4. 0.5% agarose preparation**

Timing: 45 min + overnight stirring

Conditions: RT, sterile

1. To prepare 150 mL of a 0.5% agarose solution, measure 150 mL of distilled H_2_O and add it to an autoclavable container.

2. Add a large magnetic stir bar to the container.

3. Weigh out 750 mg of Froggarose and slowly add it to the container with distilled H_2_O.

4. Autoclave the container for 30 min at 121 °C.

5. Place the container on a stir plate and continuously stir at ~700 rpm while it cools down.

6. Leave the solution on the stir plate overnight to cool.

7. Store the solution in the fridge (4 °C) until required.

**E. Preparation for bioprinting**

Timing: 2–4 h

Conditions: RT, sterile

An additional movie file shows this in more detail (see Video 1).

**Video 1.**
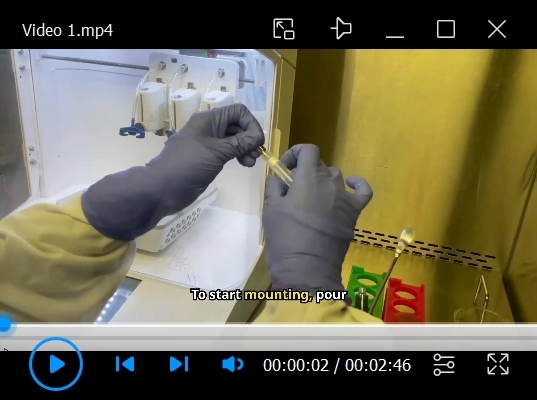
BIOX extrusion bioprinting of constructs

**E1. Autoclave the appropriate equipment**

*Note: It is recommended to do this the day before print day.*

**Figure 3.**
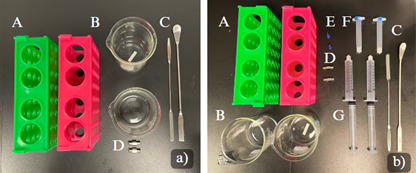
Equipment necessary for bioprinting. Autoclave all the equipment shown in A. Materials to be sterilized under UV light inside the BSC for Bioprinting are shown in B. (A) Conical racks (2×); (B) 200 mL beaker (2×); (C) spatulas (2×); (D) metal Luer locks (2×); (E) 22G, blunt needles (2×); (F) 3 mL empty cartridges with end and tip caps (2×); (G) 5 mL syringe w/o needle.

1. Autoclave all the equipment shown in Figure 3A.

2. After autoclaving, transfer the equipment (shown in Figure 3B) into the BSC.

**E2. Sterilizing the BIO X**

1. Place the BIO X printer into a sterile BSC and thoroughly decontaminate both the printer and the BSC.

2. Turn on the BIO X printer and run a UV sterilization cycle within the printer.

**Critical:** Ensure the room is vacated while the UV sterilization cycle is running.

3. After the UV sterilization of the BIO X chamber is complete, perform a UV sterilization of the entire biosafety cabinet.

**Critical:** Use UV protective glasses.

**E3. Preparing the bioink for printing**

1. Pour the prepared bioink into a sterile 10 mL syringe, ensuring it reaches the bottom.

*Note: Use a spatula if necessary.*

2. Attach the 10 mL syringe containing the bioink to an empty 3 mL cartridge using a sterilized Luer lock.

See Troubleshooting 4.

3. Load the bioink into the 3 mL cartridge by inserting the plunger into the 10 mL syringe and slowly pushing the bioink into the cartridge.

*Note: Hold the 5 mL syringe upright to allow bubbles to rise to the top, preventing them from being transferred into the pneumatic syringe.*

4. Unscrew the pneumatic syringe from the Luer lock and attach a 22G needle tip to the end.

5. Lower the printhead on the BIO X and insert the pneumatic syringe into the printhead.

6. Screw the top of the pneumatic syringe into the tube connected to the pressure connector.

**E4. Printer settings and calibration**

1. Turn on the BIO X printer and press *Bioprint*.

2. Select the desired print file.

**Figure 4.**
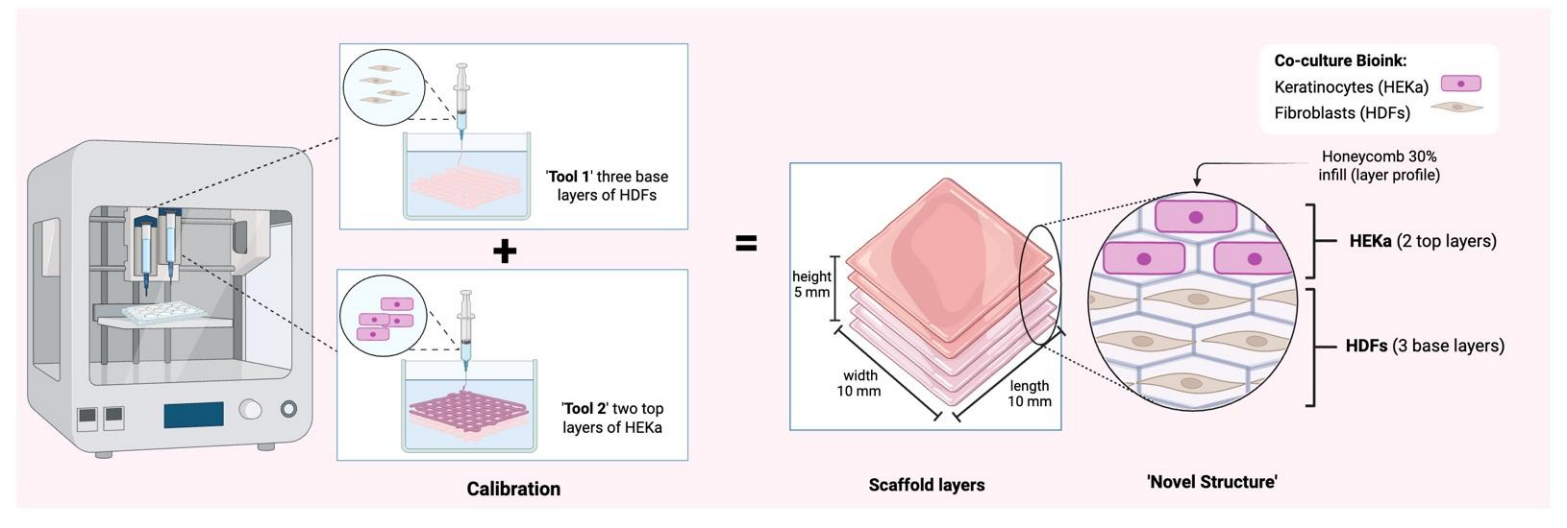
3D bioprinted co-culture skin model construct dimensions. The diagram illustrates the sequential calibration and 3D bioprinting process for a multi-layered tissue scaffold. Two distinct bioinks containing HDFs and HEKa are utilized to construct a precise *novel structure*, with HDFs forming three base layers and HEKa forming two top layers, along with a specified honeycomb infill pattern. Created in BioRender. Willerth, S. (2025) https://BioRender.com/u98s332

*Note: We printed constructs with 10 × 10 × 5 mm dimensions. Refer to Figure 4 for the characteristics of the 3D skin construct.*

3. Press the *Surface* tab at the bottom of the screen and choose a 6-well plate as the printing surface.

4. Under the *Printer* tab, select the pneumatic 3 mL option under Tool 1 and Tool 2, and select the desired pressure and speed parameters.

**Example:** Start with a speed of 15 mm/s and a pressure of 11 kPa.

5. Under the *Layers* section, upload the desired layer profile (perimeter) to print with, and select the desired infill.

6. Define the layers that are going to be printed with Tool 1 and Tool 2 by alternating the perimeter.

**Example:** Select *Honeycomb* as the layer profile with a 30% infill.

7. Under *Perimeter* and *Infill*, select the desired printheads for each printing parameter.

*Note: Select alternating* Tool 1 *to print specific layers with it, then select alternating* Tool 2 *to define the layers you want to print with it. The same applies to* Tool 3.

**Example:** Tool 1 and Tool 2 were used in our bioprinted skin model to replicate the native skin layers. Tool 1 was used to extrude the dermal layer, primarily composed of fibroblasts, forming the base of the construct. Tool 2 was used to extrude the epidermal layer (primarily composed of keratinocytes) by depositing the bioink on top of this base.

8. Test extrusion of each printhead, adjusting the pressure as needed.

9. Press *Print* and place the 6-well plate onto the print bed.

10. Select *Calibrate* and calibrate each printhead in the desired well.

*Note: Ensure both needle tips are calibrated to the same spot in the well for optimal printing results.*

**E5. Bioprinting steps**

1. Fill the 6-well plate with an agarose support bath.

*Note: Add approximately 2 mL of agarose, which should fill just over half of the well.*

**Critical:** The agarose support bath stabilizes the bioink during extrusion, enabling consistent and precise scaffold formation.

2. Test extrusion and adjust pressure.

*Note: Test extrusion of the pneumatic syringes and adjust pressure as needed.*

Recommended speed and pressure: 15 mm/s and 11 kPa.

*Note: Optimal print speed and pressure are crucial for achieving uniform printed layers and preserving cell viability. High speeds can compromise structure, while excessive pressure depletes bioink quickly and increases cell-damaging shear stress.*

3. Press *Start* to begin the printing process. See Video 1 to visualize the printing process.

4. Check speed and pressure parameters while printing, adjusting them as necessary.

5. Once the print is finished, remove the 6-well plate from the print bed.

6. Fill the 10 mL syringe fitted with a 22G needle with crosslinker and inject it slowly around the construct in a circle, careful not to touch the construct with the needle.

*Note: It is recommended to use 2–5 mL of crosslinker to fully immerse the construct.*

**Critical:** Allow the crosslinker to sit for 20–30 min.

**Figure 5.**
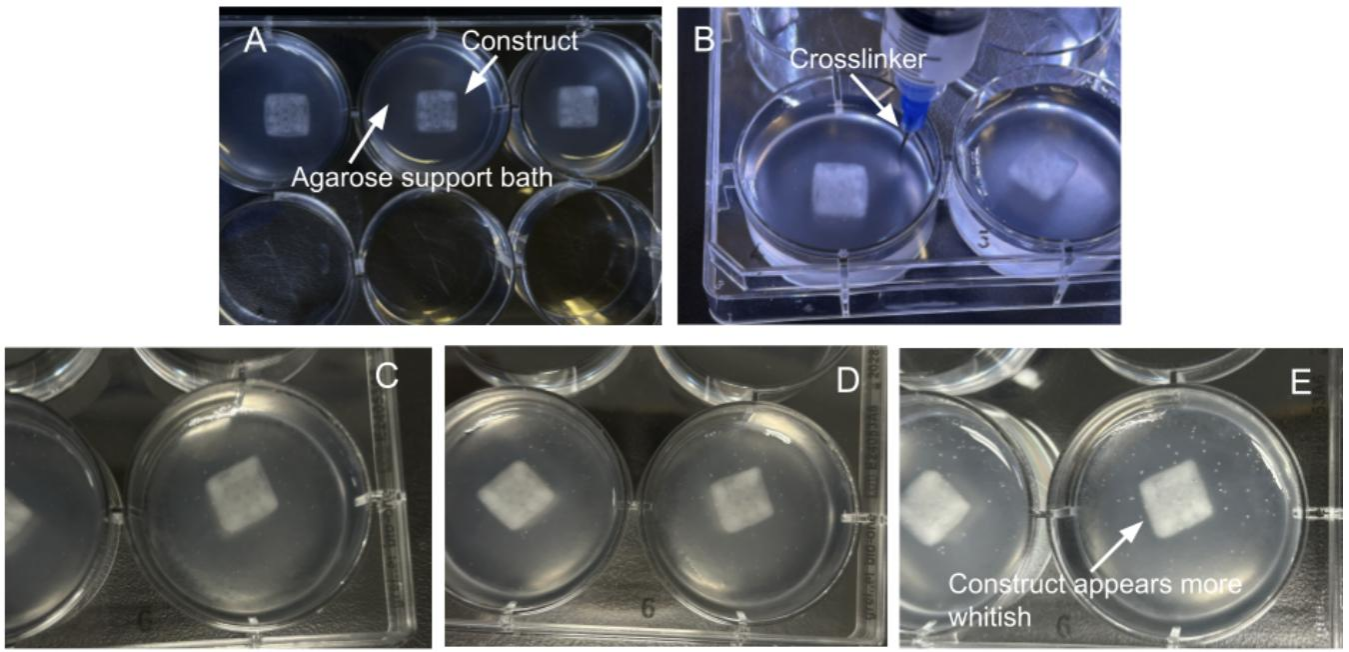
Bioprinted constructs during and after the crosslinking process. (A) Construct immediately after printing. (B) Crosslinker solution being added to the well. (C) 15-min post-crosslinker addition. (D) 25-min post-crosslinker addition, showing increased opacity. (E) 35-min post-crosslinker addition; the construct appears opaquer/whitish.

*Note: The crosslink is complete when the construct holds its shape. The appearance of the construct will be a more whitish color. Refer to Figure 5 to visualize the distinct difference between crosslinked and non-crosslinked constructs.*

7. Add TBS to a separate empty 6-well plate.

*Note: Ensure that the TBS is sterile by filtering it through a 0.2 μm filter before use.*

8. Transfer the construct using a metal spatula to the 6-well plate with sterile TBS to rinse away excess agarose.

*Notes:*

*1. To homogeneously remove excess agarose without damaging the bioprinted construct, carefully use a spatula to agitate the surrounding liquid in uniform circles near the dish wall.*

*2. The construct is not attached to the plate and should lift off easily with a metal spatula. Gently slide the edge of the metal spatula underneath the construct to loosen it and avoid using excessive force. Using two metal spatulas to support both ends of the construct can help prevent tearing or deformation.*

9. Transfer the construct to a 12-well plate containing customized media.

**Figure 6.**
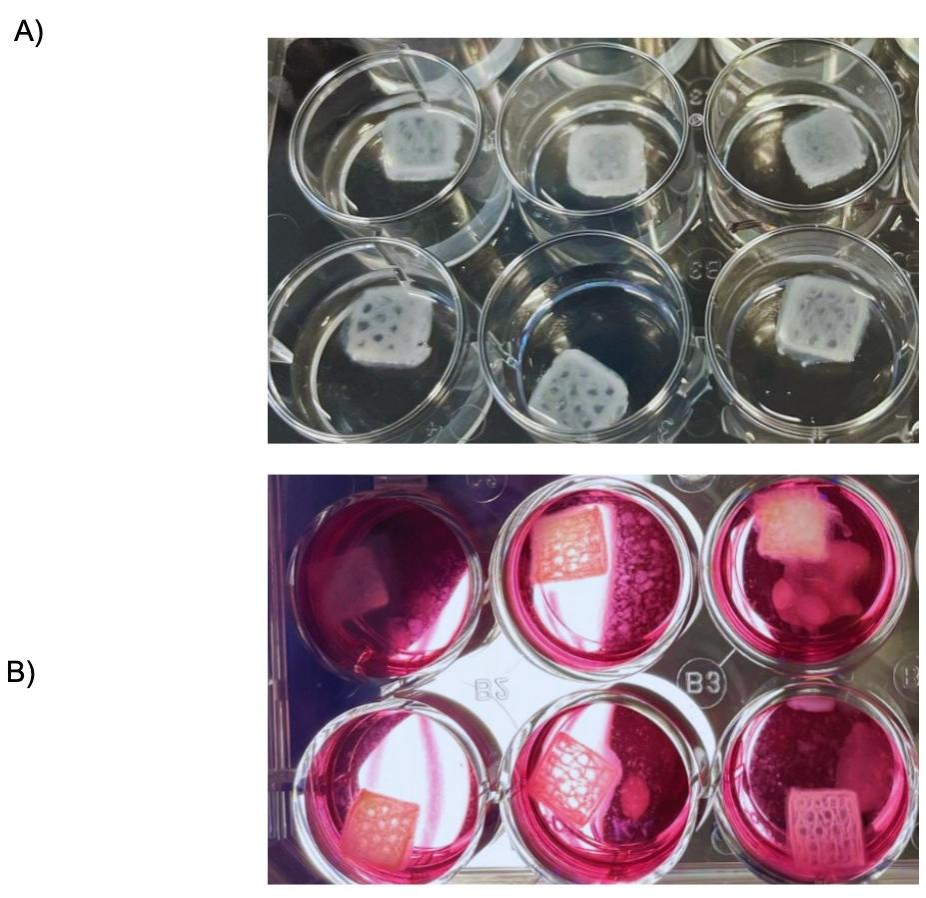
Bioprinted skin constructs. (A) 3D bioprinted skin constructs featuring a honeycomb and 30% infill density. The bottom three layers were bioprinted using a bioink containing human dermal fibroblasts (HDFs), while the upper two layers were bioprinted with a bioink containing human epidermal keratinocytes (HEKa). The constructs were placed on a 0.5% agarose bed and allowed to fully crosslink. (B) The 3D bioprinted skin constructs, after being rinsed with Tris-Buffered Saline (TBS), were transferred to a customized media well plate to facilitate cell proliferation within the constructs.

*Note: Ensure there is enough custom media in the well to completely cover the construct. Refer to Figure 6.*

**Figure 7.**
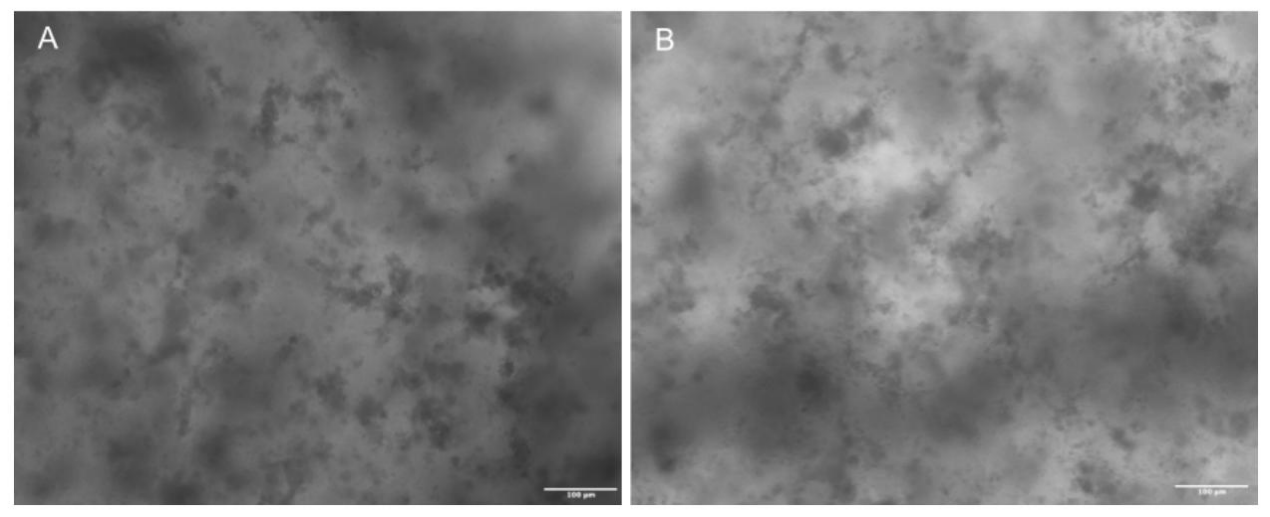
Representative images (A and B), taken with a Biotek Cytation 5 at 4× resolution, illustrate the morphology of the bioprinted cellular scaffolds. Dark spots are discernible, but their positive identification as cells requires further targeted assays. Scale bars: 100 μm.

10. Incubate the obtained constructs at 37 °C with 5% CO_2_ until bacterial infection. Refer to Figure 7 to observe the brightfield appearance of a 3D bioprinted construct.

*Note: It is recommended to proceed with the inoculation of bacteria within 48 h.*

See Troubleshooting 5.

**F. Inoculation of bacteria into the construct**

Timing: 2–3 h procedure time, three overnight (or multiple-day) incubations

Conditions: RT, sterile

**F1. Bacterial culture preparation**

Timing: Overnight + 4 h

Conditions: RT, sterile

1. Pick a single bacterial colony from a tryptic soy agar (TSA) plate.

2. Grow the colony in 5 mL of TBS overnight with shaking at 200 rpm (16–18 h).

3. Add 1 mL of overnight culture into 50 mL of sterile tryptic soy broth (TSB) in a 250 mL conical flask and incubate for 4 h at 37 °C with shaking.

4. Harvest 10 mL of culture and pellet the bacteria by centrifugation at 4,000× *g* for 7 min.

5. Wash the bacterial pellet with 1× PBS and resuspend it in 10 mL of 1× PBS.

6. Adjust the optical density at 600 nm (OD_600nm_) of the culture to 0.1 using a spectrophotometer.

**F2. Inoculation of the constructs**

Timing: 30 min

Conditions: Sterile

1. Inject the constructs with 50 μL of *S. aureus*, 50 μL of *S. epidermidis*, or both using a 27 G × 3/4” needle with a 1 mL syringe. Aim to inject into the center of the construct, approximately halfway through its depth.

**Example:** An OD of 0.1 corresponds to 5 × 10^8^ CFU/mL. For our infection model, we add a total of 5 × 10^7^ CFU per construct, comprising 2.5 × 10^7^ CFU of S. *aureus* and 2.5 × 10^7^ CFU of *S. epidermidis* (as co-infection). This translates to approximately 3.33 × 10^8^ CFU/cm^3^, based on the 150 μL volume of our standard bioprinted construct.

2. Incubate the constructs at 37 °C with 5% CO_2_ for 24 h or 72 h.

*Note: The incubation duration (e.g., 24 h, 72 h, or other time points) should be chosen based on the specific experimental objectives and the kinetic requirements of the study.*

**F3. Post-inoculation procedure**

Timing: 1 h

Conditions: Sterile

1. After incubation, aseptically remove each construct with forceps and rinse once in 1× PBS.

2. Homogenize the tissue construct in 1 mL of 1× PBS + 0.1% Triton X-100 through a 100 μm cell strainer.

*Note: While a 1 mL syringe plunger is suggested for its convenience as a sterile, flat surface, any other instrument with a similar flat, broad shape that can be easily sterilized can serve as a suitable alternative. The key is to effectively press and grind the construct to ensure complete mechanical dissociation of both the bioprinted scaffold and encapsulated cells.*

3. Collect 1 mL of the homogenate for bacterial enumeration.

**F4. Bacterial enumeration**

Timing: 2 h

Conditions: Sterile

**Figure 8.**
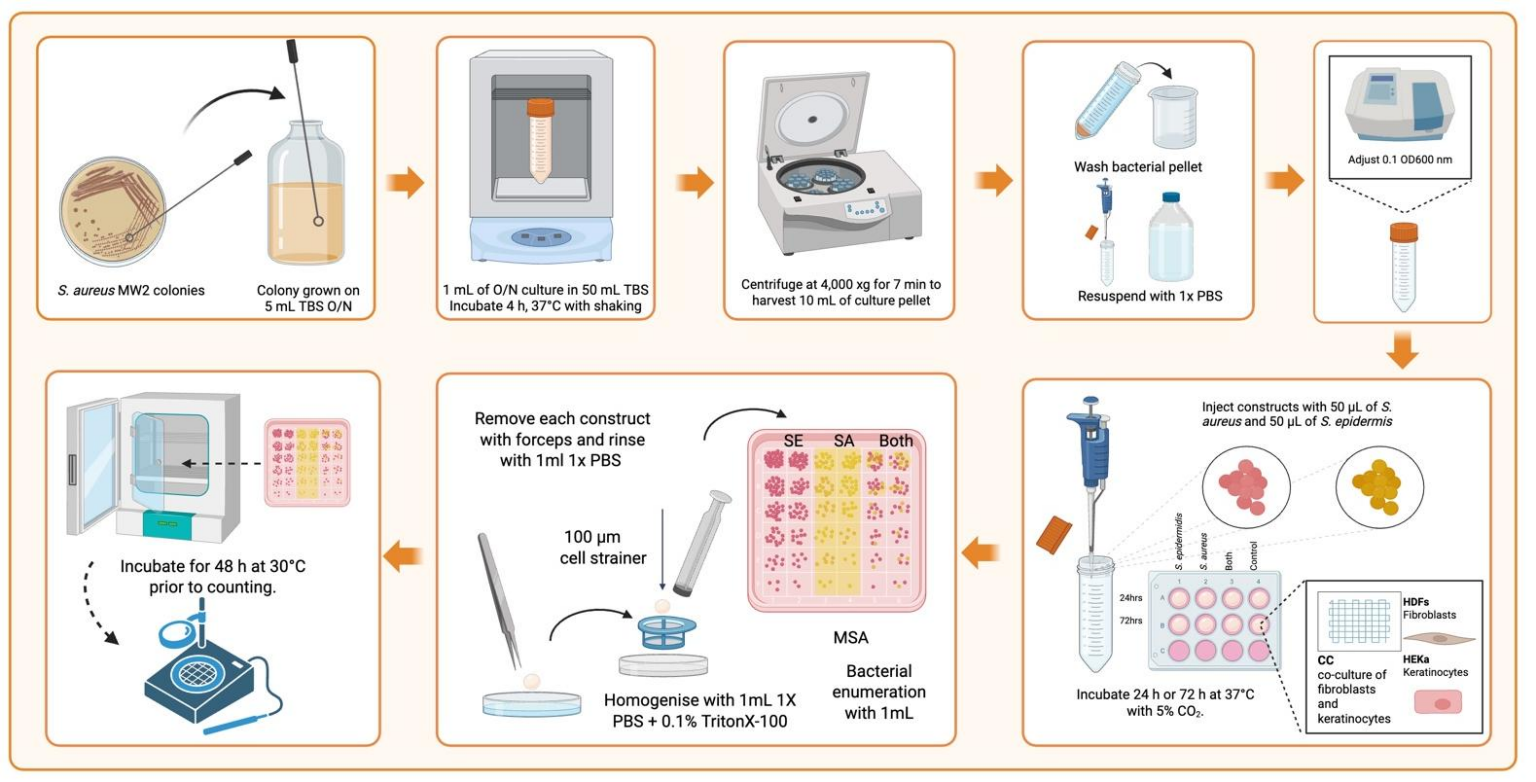
Workflow for bacterial inoculation and quantification in 3D-bioprinted tissue constructs. This diagram illustrates the sequential steps for assessing bacterial interactions with 3D-bioprinted tissue scaffolds. Key stages include bacterial culture preparation, precise injection into HDF/HEKa co-culture scaffolds, controlled incubation, and a robust recovery method involving mechanical dissociation and chemical lysis of host cells, followed by bacterial enumeration on specialized media. Created in BioRender. Willerth, S. (2025) https://BioRender.com/u98s332

1. Enumerate the bacteria using (10-fold) serial dilutions, typically up to 10^-8^, and plate 10 μL of each dilution onto mannitol salt agar (MSA) (BD) in duplicate. *S. aureus* colonies will appear yellow, and *S. epidermidis* will appear pink.

2. Incubate plates for 48 h at 30 °C prior to counting. Bacteria recovered from the construct can be prepared to determine the relative competition between the strains in the construct. Refer to Figure 8 for a step-by-step overview of the bacterial inoculation and quantification process in the 3D construct.

## Data analysis

While *S. epidermidis* can act as an opportunistic pathogen itself, it more often behaves as a benign or beneficial member of the skin microbiota, involved in barrier development, maintenance of homeostasis, and control of opportunistic pathogens, particularly antagonizing *S. aureus* proliferation [30].

*S. epidermidis* produces molecules known as phenol-soluble modulins, which exhibit antimicrobial activity against pathogens such as *Streptococcus pyogenes* and *S. aureus* [31].

**Figure 9.**
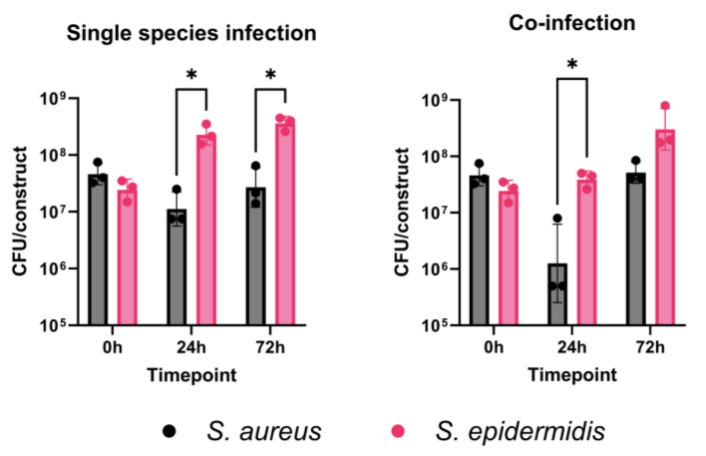
3D-bioprinted skin constructs can be used to model interspecies competition in the skin. Individual constructs were infected with 3–5 × 10^7^ CFU (colony forming units) of *Staphylococcus aureus* MW2, *Staphylococcus epidermidis* RP62a, or both and incubated for 24 or 72 h at 37 °C with 5% CO_2_. Constructs were homogenized with 1 mL of 1× PBS + 0.1% Triton X-100 and then plated on mannitol salt agar (MSA) (BD) in duplicate. Plates were incubated for 48 h at 30 °C and then enumerated. Points represent individual infections; bars represent the geometric mean ± geometric standard deviation. Significant differences were determined using an unpaired T-test (*p < 0.05).

Therefore, we expect that *S. aureus* will not proliferate as extensively as *S. epidermidis* in individual species infections within the skin constructs. Similarly, we anticipate that *S. epidermidis* will outcompete *S. aureus* in the co-infection skin model, replicating behavior observed in human skin. Using this model, we demonstrated that *S. aureus* does not grow as well as *S. epidermidis* in the skin construct during single-species infections. This difference becomes more pronounced during co-infection, especially at 24 h post-infection (Figure 9).

## Validation of protocol

The 3D bioprinted co-culture skin model has been validated in the following research article:

• Andrade et al. [17]. 3D Bioprinting a Novel Skin Co-Culture Model Using Human Keratinocytes and Fibroblasts. *Journal of Biomedical Materials Research Part A.* 113(1), e37831. https://doi.org/10.1002/jbm.a.37831

Our study expands this model by incorporating bacterial co-infection to examine host–pathogen interactions in a more physiologically relevant environment. Based on these outcomes, we foresee that the 3D-bioprinted constructs will serve as a feasible model for studying more complex bacterial co-infections and interactions between bacteria and skin cells. In conclusion, we expect our findings to indicate that 3D-bioprinted skin constructs can effectively mimic in vivo bacterial interactions and provide valuable insights into infection dynamics.

## General notes and troubleshooting

**General notes**

1. Allowing the printed constructs to mature for at least 24–48 h before bacterial inoculation will support increased epidermal maturation and structural stability, enhancing experimental outcomes.

2. While CFU enumeration is used in this protocol, complementary methods such as qPCR or fluorescent imaging could provide additional insights into bacterial localization and/or gene expression within the construct.

3. The protocol can be adapted for other cell types or bacterial species, but adjustments may be necessary for cell seeding densities, print parameters, or bacterial inoculation conditions. Conduct pilot experiments when adapting the protocol to new models.

4. The fibrin-based bioink undergoes natural degradation, potentially limiting the duration of skin–microbe interaction studies. This may affect the structural integrity of the model and influence experimental outcomes over extended periods.

5. Printed constructs should be stored in the appropriate media at 37 °C with 5% CO_2_ and monitored for degradation over time. If extended culture is required, optimizing media formulations to support epidermal maturation may be necessary.

**Troubleshooting**

Problem 1: Cells do not adhere to the flask during cell seeding.

Possible cause: Both HDFs and HEKa are temperature-sensitive and may not adhere properly if conditions are not optimal.

Solution: Ensure that all reagents or solutions are at room temperature before use, and prewarm the medium in the flasks to 37 °C before seeding or passaging the cells.

Problem 2: Cells do not detach completely from the flask during passaging.

Possible cause: The trypsin-EDTA is either not at the optimal temperature or has passed its optimal usage period.

Solution: Place the flasks with 1 mL of Trypsin-EDTA in the incubator for 5 min to enhance the detachment of cells. It is also recommended to use fresh trypsin-EDTA or one that has not been thawed for more than a week.

Problem 3: Bubbles form when mixing the bioink components.

Possible cause: Some of the bioink components are quite viscous and prone to generating bubbles when mixed.

Solution: Mix the components carefully with a spatula or a pipette. If bubbles form, aspirate them with a pipette to prevent disruption in the bioink.

Problem 4: Bioink is lost when transferring from the 50 mL conical to the CC Luer lock syringe without a needle.

Possible cause: The viscous consistency of the bioink causes it to stick to the walls of the conical, leading to loss during transfer.

Solution: Clean the 50 mL conical as thoroughly as possible using a round spatula to reduce any bioink residue left on the walls.

Problem 5: If, during printing, the bioink is no longer being extruded and there is still some left in the cartridges, the 22G blunt needles may be clogged.

Solution: It is recommended to replace with a fresh 22G blunt needle and readjust the pressure.
